# Promotion of Flowering by *Apple Latent Spherical Virus* Vector and Virus Elimination at High Temperature Allow Accelerated Breeding of Apple and Pear

**DOI:** 10.3389/fpls.2016.00171

**Published:** 2016-02-25

**Authors:** Norioko Yamagishi, Chunjiang Li, Nobuyuki Yoshikawa

**Affiliations:** Plant Pathology Laboratory, Faculty of Agriculture, Iwate UniversityMorioka, Japan

**Keywords:** *Apple latent spherical virus*, viral vector, apple, pear, promotion of flowering, elimination of virus

## Abstract

Plant viral vectors are superior tools for genetic manipulation, allowing rapid induction or suppression of expression of a target gene in plants. This is a particularly effective technology for use in breeding fruit trees, which are difficult to manipulate using recombinant DNA technologies. We reported previously that if apple seed embryos (cotyledons) are infected with an *Apple latent spherical virus* (ALSV) vector (ALSV-AtFT/MdTFL1) concurrently expressing the *Arabidopsis thaliana* florigen (*AtFT*) gene and suppressing the expression of the apple *MdTFL1-1* gene, the period prior to initial flowering (generally lasts 5–12 years) will be reduced to about 2 months. In this study, we examined whether or not ALSV vector technology can be used to promote flowering in pear, which undergoes a very long juvenile period (germination to flowering) similar to that of apple. The *MdTFL1* sequence in ALSV-AtFT/MdTFL1 was replaced with a portion of the pear *PcTFL1-1* gene. The resulting virus (ALSV-AtFT/PcTFL1) and ALSV-AtFT/MdTFL1 were used individually for inoculation to pear cotyledons immediately after germination in two inoculation groups. Those inoculated with ALSV-AtFT/MdTFL1 and ALSV-AtFT/PcTFL1 then initiated flower bud formation starting one to 3 months after inoculation, and subsequently exhibited continuous flowering and fruition by pollination. Conversely, Japanese pear exhibited extremely low systemic infection rates when inoculated with ALSV-AtFT/MdTFL1, and failed to exhibit any induction of flowering. We also developed a simple method for eliminating ALSV vectors from infected plants. An evaluation of the method for eliminating the ALSV vectors from infected apple and pear seedlings revealed that a 4-week high-temperature (37°C) incubation of ALSV-infected apples and pears disabled the movement of ALSV to new growing tissues. This demonstrates that only high-temperature treatment can easily eliminate ALSV from infected fruit trees. A method combining the promotion of flowering in apple and pear by ALSV vector with an ALSV elimination technique is expected to see future application as a new plant breeding technique that can significantly shorten the breeding periods of apple and pear.

## Introduction

In woody fruit trees, the duration from seed germination to flowering (juvenile period) is long. During this time, plants undergo only vegetative growth, with no flowering or fruition. Thus, breeding new cultivars of plants such as apples and pears can require several dozen years (Crosby et al., 1992, 1994; Fischer, 1994; Flachowsky et al., 2009; Freiman et al., 2012). For that reason, shortening the juvenile period is key to increasing the efficiency of fruit tree breeding. A great deal of information on genes that direct plant flowering has been accumulated during the past 20 years. The best-known is the *FLOWERING LOCUS T* (*FT*) gene, which encodes the flowering hormone florigen; it was designated by Chailakhyan as the specific substances which form the flower by floral stimulus in 1936 (Zeevaart, 2006). The *FT* gene encodes an approximately 20 kDa water-soluble protein that was first reported in *Arabidopsis thaliana* (*AtFT*) and rice (*Heading date3a, Hd3a*) (Corbesier et al., 2007; Tamaki et al., 2007), and belongs to the phosphatidylethanolamine binding protein family (Kardailsky et al., 1999; Kobayashi et al., 1999). This family is conserved across all angiosperm species, (Hiraoka et al., 2008). Recombinant DNA technology has been used to overexpress citrus *CiFT* in orange and pear, and apple *MdFT* in apple, leading to the induction of early flowering (Endo et al., 2005; Matsuda et al., 2009; Kotoda et al., 2010; Tränkner et al., 2010). The phosphatidylethanolamine binding protein family genes regulating flowering include *TERMINAL FLOWER 1* (*TFL1)* in *A. thaliana*. The *TFL1* gene is highly homologous to the *FT* gene, but performs the opposite function: it suppresses flowering (Bradley et al., 1997; Boss et al., 2004; Hanzawa et al., 2005). Fruit trees also possess genes homologous to the *TFL1* gene (Yao et al., 1999; Kotoda et al., 2002; Esumi et al., 2005; Mimida et al., 2009; Freiman et al., 2012). Suppression of apple *MdTFL1* and pear *PcTFL1-1* and *PcTFL1-2* expression reportedly induces early flowering in both plants (Kotoda et al., 2006; Flachowsky et al., 2012; Freiman et al., 2012). Because the genes involved in flowering have now been identified, it is becoming possible to shorten the juvenile period via manipulation of these genes. However, recombinant DNA technologies require a relatively long culture and plant regeneration period, and are applicable to a limited number of species and varieties. In addition, field cultivation of genetically modified plants is tightly restricted in countries such as Japan, where they are regulated by the Act on the Conservation and Sustainable Use of Biological Diversity through Regulation of the Use of LMOs (Cartagena Protocol).

A viral vector system is one DNA recombination method used to control gene expression and suppression. *Apple latent spherical virus* (ALSV) is a spherical virus with a diameter of approximately 30 nm, composed of two RNA genome segments (RNA1 and RNA2) and three coat proteins (Vp25, Vp20, and Vp24) (Li et al., 2000). Although its only natural host is apple, in experimental settings, ALSV infects *Solanaceae*, *Cucurbitaceae*, *Fabaceae*, and fruit trees in the *Rosaceae* (including apple); the infection is usually asymptomatic. We have constructed an ALSV vector in which a site for foreign gene insertion has been added to the RNA2 genome of ALSV, and determined that the ALSV vector system can be used to induce foreign gene expression, virus-induced gene silencing, and virus-induced transcriptional gene silencing over a long period of time and in various plant species (Li et al., 2004; Igarashi et al., 2009; Yamagishi and Yoshikawa, 2009, 2011; Sasaki et al., 2011; Takahashi et al., 2013; Taki et al., 2013; Tamura et al., 2013; Kon and Yoshikawa, 2014; Satoh et al., 2014; Yamagishi et al., 2014; Nakatsuka et al., 2015). Additionally, ALSV vectors can concurrently induce and suppress the expression of two types of genes (Yamagishi et al., 2014). We reported that the inoculation of apple cotyledons immediately after germination with an ALSV vector (ALSV-AtFT/MdTFL1) concurrently expressing the *AtFT* gene and suppressing expression of the *MdTFL-1-1* gene can shorten the period from seeding to flowering, which usually lasts 5–12 years, to approximately 2 months after germination. In addition, this process can shorten generation times in order to obtain next-generation seeds in 1 year or less (Yamagishi et al., 2014). Because ALSV vectors carrying a foreign gene are subject to regulation by the Cartagena Protocol, as are genetically modified plants, development of a method for eliminating ALSV vectors from infected plants is necessary. We reported that most next-generation seedlings obtained from ALSV vector-infected plants become free of the virus (Nakamura et al., 2011; Kishigami et al., 2014; Yamagishi et al., 2014). Thus, they can be considered non-genetically modified plants and be used as breeding stock (Yamagishi et al., 2014). However, when the ALSV-vector-infected plant is itself a promising breeding stock, a method of eliminating ALSV vectors from the infected plant would make ALSV vector technology even more useful.

In this article, we report our study of the ALSV vector-facilitated promotion of flowering in pear, which has a long juvenile period. In addition, we describe a simple method of eliminating ALSV vectors from infected apple and pear plants. Together, these procedures constitute a new plant breeding technique that significantly shortens the period necessary for breeding, and contributes to efficient selective breeding.

## Materials and Methods

### Plants

*Nicotiana benthamiana* was used for agro-inoculation of binary vectors and *Chenopodium quinoa* was used for virus propagation (Kon and Yoshikawa, 2014; Yamagishi et al., 2014).

For inoculation of ALSV vectors to apple, pear, and Japanese pear, seeds of apple ‘Ourin,’ pear ‘La France,’ and ‘Bartlett,’ and Japanese pear ‘Shinkou,’ which had been incubated at 4°C for more than 3 months, were used.

### Construction of ALSV Vectors

**FIGURE 1:**
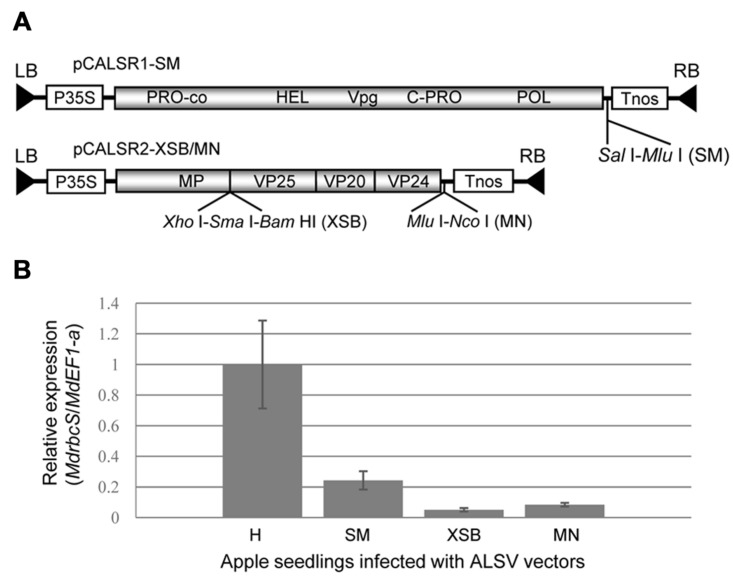
**Schematic representation of *Apple latent spherical virus* (ALSV) binary vectors and comparison of their capability to induce RNA silencing at their three cloning sites.** **(A)** Genetic map of ALSV binary vector (pBCALR1-SM and pBCALR2-XSB/MN). LB and RB, left and right borders, respectively; P35S, CaMV 35S RNA promoter; NOS, nopaline synthase terminator. The open reading frames of ALSV represent the protease co-factor (Pro-co), NTP-binding helicase (HEL), cysteine protease (C-Pro), RNA polymerase (POL), movement protein (MP), and three capsid proteins (Vp25, Vp20, and Vp24). **(B)** Comparison of RNA silencing efficiency for the three cloning sites of ALSV vectors with a fragment of the *MdrbcS* gene inserted. The relative expression of *MdrbcS*-mRNA was estimated by quantitative RT-PCR.

In this study, we used the binary vectors pCALSR1 and pCALSR2-XSB/MN described by Kon and Yoshikawa (2014) and another binary vector (pCALR1-SM) with a cloning site (*Sal*I-*Mlu*I) inserted into the 3′ non-coding region of ALSV-RNA1 (**Figure 1A**).

To compare the capability to induce RNA silencing among the three cloning sites in the ALSV vector shown in **Figure 1A**, a DNA fragment (201 bp) of *MdrbcS*-mRNA (accession no. L24497; nt 50–250) was amplified from an *MdrbcS*-cDNA clone (Sasaki et al., 2011) using primer pairs with restriction enzyme recognition sequences for cloning to all three sites. The DNA products were double-digested with enzymes (*Sal*I plus *Mlu*I, *Xho*I plus *Bam*HI, or *Mlu*I plus *Nco*I) and ligated to pCALSR1-SM and pCALSR2-XSB/MN digested with the same enzymes.

Full-length DNA of *AtFT* (accession no. AB027504; Yamagishi et al., 2011), a partial sequence of *MdTFL1-1* (accession no. AB052994; nt 40-223; Sasaki et al., 2011), and *PcTFL1-1* (accession no. AB162042; nt 195-400) were inserted into pCALSR2-XSB/MN (full-length DNA of *AtFT* and partial sequence of *MdTFL1-1*) and pCALSR1-SM (containing either partial sequence of *MdTFL1-1* or *PcTFL1-1*).

### Agro-Inoculation

For agro-inoculation, the pCALSR1- and pCALSR2-based binary vectors described above were transformed into *Rhizobium radiobacter* strain GV3101::pMP90, respectively. Agro-inoculation was carried out as described by Kon and Yoshikawa (2014). The resulting viruses for induction of early flowering were designated ALSV-AtFT, ALSV-AtFT/MdTFL1, ALSV-AtFT/PcTFL1, and ALSV-AtFT/MdTFL1/R2.

### Preparation of Viral Inocula

Sap of agro-inoculated *N. benthamiana* leaves was inoculated into *C. quinoa* by rubbing them with carborundum. ALSV vectors were purified from infected *C. quinoa* leaves by homogenizing with 0.1 M Tris buffer (pH 7.8), clarifying with bentonite, and precipitating with PEG 6000 as described previously (Li et al., 2000). ALSV-RNAs were extracted by phenol/chloroform and then precipitated with ethanol (Yamagishi et al., 2010).

### Inoculation of ALSV-RNAs by Particle Bombardment

Cotyledons of apple, pear, and Japanese pear just after germination were biolistically inoculated with ALSV-RNAs according to the method reported by Yamagishi et al. (2010) with slight modification. Biolistic inoculation was conducted at a pressure of 300 psi (Helios Gene Gun system; Bio-Rad) or 1100 psi (PDS-1000/He Particle Delivery System; Bio-Rad) using helium gas and with two shots per cotyledon.

For inoculation of apple leaves, seedlings at the 2, 3, and 4 leaf stages were biolistically inoculated with ALSV-RNAs (1 shot per leaf) to all true leaves at a pressure of 100 psi (Helios Gene Gun system).

In experiments on elimination of virus, *Apple chlorotic leaf spot virus* (ACLSV) was used as a control virus. A mixture of ALSV-RNAs and ACLSV-RNA was biolistically inoculated to apple cotyledons just after germination as described above, and the infected plants were incubated in a growth chamber at 25°C (day length of 16 h) for about 3 months. Pear cotyledons were inoculated with ALSV-RNAs similarly.

### High Temperature Treatment

Infected apple and pear seedlings (7–10 leaf stage) grown in a growth chamber at 25°C were moved into a growth chamber at 37°C and incubated for 4 weeks. After that, the plants were transferred to a green house at 25°C and grown until analyzed.

### Quantitative Real-Time PCR (qRT-PCR)

Quantitative RT-PCR for evaluation of relative expression levels of *MdrbcS*-mRNA was conducted as follows. Total RNA was extracted from apple leaves infected with the three ALSV vectors having partial sequence of *MdrbcS* according to a method reported previously (Yamagishi et al., 2014). RNA samples were treated with DNase I followed by phenol/chloroform extraction and ethanol precipitation. First-strand cDNA was synthesized using 500 ng total RNA as a template, oligo (dT) primers and ReverTra Ace reverse transcriptase (Toyobo). Quantitative RT-PCR was performed in a reaction volume of 20 μl using 2 μl cDNA, SYBR Premix Ex Taq (Tli RNase H Plus; TaKaRa) and the following primer pairs (final concentrations of 0.2 μM): MdrbcS380(+) [5′-atggaaggtactggacaatg-3′] and MdrbcS500(-) [5′-cgatgatacggatgaagg-3′] for amplification of *MdrbcS*; and MdEF1-a254(+) [5′-gatgcaggtatggtgaagat-3′] and MdEF1-a365(-) [5′-taacaccaacagcaacagtc-3′] for amplification of *MdEF1-a*. The conditions for RT-PCR performed with the Eco Real-Time PCR system (Illumina) were 95°C for 30 s followed by 40 cycles at 95°C for 5 s, annealing at 60°C for 30 s, and a final melting step up to 95°C for 15 s, 55°C for 15 s and 95°C for 15 s. Expression levels were normalized with *MdEF1-a.* Data (for five seedlings/experiment) were analyzed by 2-ΔΔCT data analysis according to User Bulletin #2: ABI PRISM 7700 Sequence Detection System.

### Detection of Viruses

RNA extraction from infected plants and RT-PCR were conducted as described before (Kishigami et al., 2014; Yamagishi et al., 2014). For ALSV detection by RT-PCR, two primer pairs, ALS 1418(+) [5′-cccaaatctgctagaaggtc-3′] and ALS 1511(-) [5′-gcaaggtggtcgtga-3′]; or ALS 999(+) [5′-gctctctgtagttattctgcag-3′] and ALS 1437(-) [5′-gaccttctagcagatttggg-3′] were used. For ACLSV detection, ACLSV CP 39-57 (+) [5′-agatctgaaagcgttcctg-3′] and ACLSV CP559-582 (-) [5′-ctaaatgcaaagatcagttgtaac-3′] were used.

The detection of ALSV by qRT-PCR was conducted as described by Kishigami et al. (2014).

Dot-blot hybridization was carried out as described by Yamagishi et al. (2006).

*In situ* hybridization was carried out as described by Nakamura et al. (2011).

## Results

### Inoculation of ALSV Vectors into Apple, Pear, and Japanese Pear

Generally, it is not easy to establish a successful viral infection in woody fruit trees. When using the ALSV vector system to infect fruit trees, it is important to have an efficient inoculation technique. We have already reported that inoculation of apple cotyledons just after germination with ALSV-RNAs using particle bombardment leads to infection with over 90% efficiency (Yamagishi et al., 2010). In this study, we compared the infection rate of seed embryos after particle bombardment inoculation of three pome fruit species (apple, pear, and Japanese pear). As shown in **Table 1**, for cotyledons inoculated immediately after germination, an RT-PCR assay of the viral RNA present 1 week later detected 100% infection for all three plant species. Next, in order to investigate whether ALSV moved and the infection spread throughout the plant, we examined viral multiplication in the true leaves that developed 1 month after inoculation (the fifth leaf in apple, and the fifth or sixth leaf in pear and Japanese pear). The virus was detected in the true leaves of 90–100% of inoculated apple seedlings, as reported previously (Yamagishi et al., 2011, 2014). In addition, 70% of pear seedlings exhibited systemic viral infection. In contrast, in Japanese pear, the virus was detected in the upper leaves of no more than around 20% of inoculated seedlings; the systemic infection rate was 0% in some experimental trials. Thus, the systemic infection rate of the ALSV vector differed among the three species, and was particularly low in Japanese pear (**Table 1**).

**Table 1:** Infection rate of *apple latent spherical virus* (ALSV) vectors to apple, pear, and Japanese pear following biolistic inoculation^∗^.

Plants	Inoculation sites (plant stages)	Infection rate (%)	
		
		Local (cotyledons)	Systemic (true leaves)
Apple	Cotyledon (seed) ^∗∗^	100	90–100
Pear	Cotyledon (seed)	100	70
Japanese pear	Cotyledon (seed)	100	0–20
Apple	True leaf (2nd leaf stage)^∗∗∗^	100	50
	True leaf (3rd leaf stage)	100	20–50
	True leaf (4th leaf stage)	100	20


*^∗^Experiments were repeated at least twice (at least six plants per experiment). wtALSV and ALSV-AtFT/MdTFL1 were used as inocula. Virus was detected by RT-PCR at 7 days post inoculation (dpi) for inoculated leaves and at 30 dpi for upper true leaves. ^∗∗^Cotyledons just after germination were inoculated with ALSV-RNAs. ^∗∗∗^First leaf at second leaf stage, first and second leaves at third leaf stage, and first to third leaves at fourth leaf stage were inoculated with ALSV-RNAs.*

We previously reported that biolistic inoculation of true leaves of apple resulted in extremely low infection rates (Yamagishi et al., 2010). In this study, we re-examined the infection rate in true leaves at different stages in apple. Use of particle bombardment with viral RNA to inoculate the first true leaf of each apple seedling at the two-leaf stage led to detection of systemic infection in 50% of plants. Inoculation of true leaves during the three- and four-leaf stages of apple resulted in 20–50 and 20% systemic infection rates, respectively (**Table 1**). Thus, inoculation of true leaves is possible up to the four-leaf stage, although the infection rate declines as the growth stage advances (**Table 1**).

Based on these results, we selected cotyledon stage for infection in apple and pear, and used apple and pear for the experiments on promotion of flowering and virus elimination.

### Construction of ALSV Vectors Expressing *ATFT* and Suppressing *TFL* Genes Concurrently

We have already reported on the construction of a binary vector (pBCALR2-XSB/MN), in which ALSV genomic RNAs were inserted into the binary vector pCAMBIA1300, and a new cloning site (*Mlu*I-*Nco*I) was added to the 3′ non-coding region of the ALSV-RNA2 (**Figure 1A**; Kon and Yoshikawa, 2014). In this study, we constructed another binary vector (pBCALR1-SM) into which a cloning site (*Sal*I-*Mlu*I) was inserted into the 3′ non-coding region of ALSV-RNA1 (**Figure 1A**). In these vectors, the target gene needs to be inserted at the XSB site in the ORF of RNA2 when aiming for gene expression. Conversely, when aiming for the suppression of gene expression by RNA silencing, the gene should be inserted into the MN site of the 3′ non-coding region of the RNA2, the SM site of the 3′ non-coding region of the RNA1, or the XSB site of the RNA2. These three cloning sites exhibited slight differences in their capacity to induce silencing, as shown by the varying efficiency of suppressing *MdrbcS*-mRNA expression in apple via silencing: XSB > MN > SM (**Figure 1B**).

We prepared ALSV vectors (ALSV-AtFT, ALSV-AtFT/MdTFL1, ALSV-AtFT/PcTFL1, and ALSV-AtFT/MdTFL1/R2) by inserting the full-length *AtFT* gene and a portion of the *TFL* gene from apple (*MdTFL1-1*) or pear (*PcTFL1-1*) into ALSV-cDNA, as described in section “Material and Methods.” ALSV-AtFT/MdTFL1, ALSV-AtFT/MdTFL1/R2, and ALSV-AtFT/PcTFL1 are vectors that express the *AtFT* gene and concurrently suppress the apple or pear *TFL* gene (*MdTFL1-1* and *PcTFL1-1*), whereas ALSV-AtFT/MdTFL1 is highly effective at promoting the flowering of apple, as previously reported (Yamagishi et al., 2014). We investigated the effect of these vectors on flowering induction.

### Effects of ALSV-AtFT, ALSV-AtFT/MdTFL1, ALSV-AtFT/MdTFL1/R2, and ALSV-AtFT/PcTFL1 on Flowering in Apple and Pear Seedlings

**Table 2** summarizes the early flowering rates of apple and pear plants infected by each of the ALSV vectors. In apple seedlings inoculated with ALSV-AtFT, early flowering occurred in 17% of infected seedlings one and a half to 2 months after inoculation. Flowering occurred only once (i.e., one flower was produced), as previously reported (Yamagishi et al., 2011). In contrast, apple seedlings inoculated with ALSV-AtFT/MdTFL1 began flowering one and a half to 2 months after inoculation, and underwent continuous flowering thereafter, with a 93% flowering rate. The apple seedlings inoculated with ALSV-AtFT/MdTFL1/R2 exhibited 100% flowering. Though two out of twelve infected seedlings exhibited continuous flowering, as did plants infected with ALSV-AtFT/MdTFL1/R2, ten out of twelve infected plants flowered only once. RT-PCR analysis of the retention of the *AtFT* gene revealed that plants with a single flower had lost the *AtFT* gene.

**Table 2:** Promotion of flowering in apple and pear seedlings by infection with *Apple latent spherical virus* vectors.

Fruit trees	ALSV vectors	Genes for		No. of flowering/infected plants (%)	Flowering characteristics
					
		Expression	Suppression		
Apple	ALSV-AtFT	*AtFT*	–	2/12 (17)	One flower
	ALSV-AtFT/MdTFL1	*AtFT*	*MdTFL1-1*	13/14 (93)	Continuous flowering
	ALSV-AtFT/MdTFL1/R2	*AtFT*	*MdTFL1-1*	12/12 (100)	One flower^∗^ or Continuous flowering
Pear	ALSV-AtFT	*AtFT*	–	0/11 (0)	–
	ALSV-AtFT/MdTFL1	*AtFT*	*MdTFL1-1*	9/27 (33)	Continuous flowering
	ALSV-AtFT/PcTFL1	*AtFT*	*PcTFL1-1*	6/7 (86)	Continuous flowering


*^∗^Most plants showing one flower lacked an FT insert in ALSV-AtFT/MdTFL1/R2 based on RT-PCR, suggesting that the inserted FT gene is unstable in ALSV-AtFT/MdTFL1/R2.*

**FIGURE 2:**
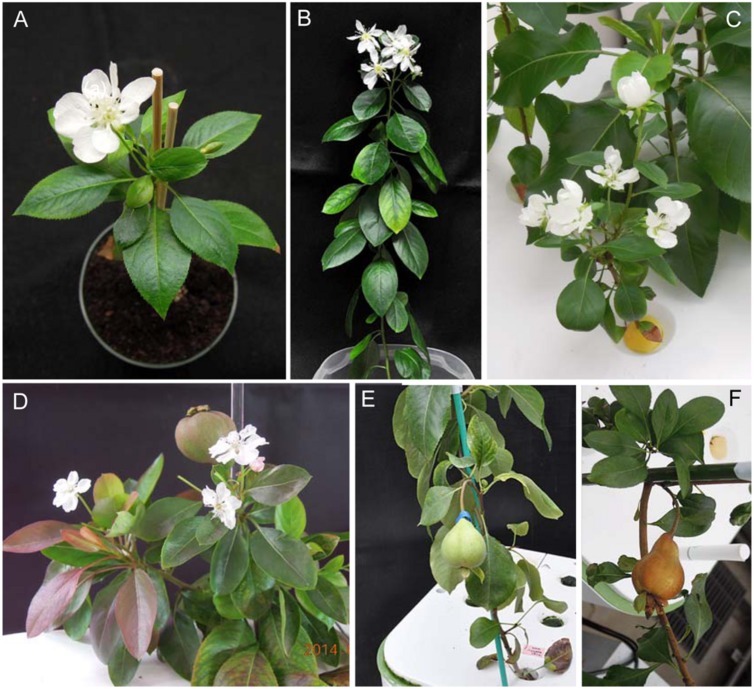
**Precocious flowering of pear seedlings infected with ALSV-AtFT/MdTFL1 **(A,C–E)** and ALSV-AtFT/PcTFL1(B,F)**.

Next, we investigated the effect of ALSV vectors on flowering promotion in pear: no early flowering was induced in plants infected with ALSV-AtFT, whereas early flowering was induced in 33% of plants infected with ALSV-AtFT/MdTFL1 and 86% infected with ALSV-AtFT/PcTFL1. As in the infected apple plants, flowering started at approximately the 15-leaf stage, one and a half to 3 months after inoculation. As in apple, plants with a single flower at the apex and plants with concurrent multiple flowering were both observed (**Figures 2A,B**), but in pear, plants with multiple flower buds forming on the lateral shoots were also observed (**Figures 2C,D**). Most flowers exhibited a normal morphology with stamens and pistils, and their fruits developed by pollination (**Figures 2D–F**).

Similarly to early flowering in apple seedlings, the ALSV vector induced flowering in pear between one and one half and three months; infected plants then flowered continuously for several months thereafter (**Figure 2D**).

### ALSV does not Move Systemically in Infected Apple or Pear Plants After Exposure to High Temperature

**FIGURE 3:**
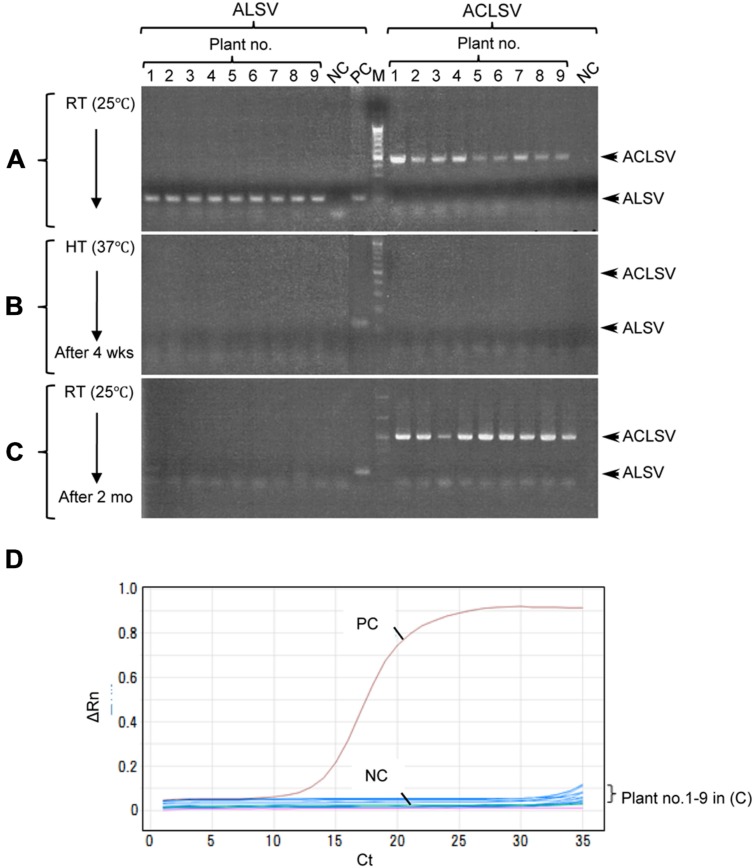
**RT-PCR detection of ALSV and ACLSV from apple seedlings before and after high-temperature treatment.** **(A–C)** Detection of ALSV and ACLSV from apple seedlings grown at 25°C **(A)**, then grown at 37°C for 4 weeks (wks; **B**), and transferred to 25°C and grown for 2 months (mo; plant nos. 1–9) in **(C)**. **(D)** Quantitative RT-PCR of ALSV from apple seedlings (plant nos. 1–9) in **(C)**.

Successful elimination of ALSV vectors from infected plants may allow, the use of early flowering plants as breeding stock without genetic modification. We have frequently observed a phenomenon in which ALSV multiplied in inoculated leaves but did not move to the upper leaves; this is particularly associated with true leaf viral inoculation (**Table 1**). Therefore, we considered the possibility of stopping the long-distance movement of ALSV in infected plants. According to the literature, to produce virus-free plants of fruit trees such as apple, shoot apex culture after growth for several weeks at high temperature (37–38°C) is generally adopted. First, we inoculated apple seedlings with ALSV and ACLSV to produce individual seedlings infected with both viruses. ACLSV, a representative apple latent virus, was used as a control virus difficult to remove only by a high temperature treatment. RT-PCR using ALSV-specific and ACLSV-specific primers detected the multiplication of both viruses in infected apple plants grown at 25°C (**Figure 3A**, **Table 3**). An RT-PCR assay of the top leaves of these plants after a high-temperature (37°C) incubation for 4 weeks detected neither of the viruses (**Figure 3B**, **Table 3**). Thus, replication of the two viruses is likely to be inhibited by high temperature. After a subsequent 2 months of growth at 25°C, examination of viral multiplication in the top leaves of high temperature-treated apple seedlings detected no ALSV in any plants. Conversely, ACLSV began to replicate again in all plants (**Figure 3C**, **Table 3**). No ALSV multiplication was detected in any samples, even in a quantitative RT-PCR assay of RNA collected from the apple plants in **Figure 3C**, a test with greater sensitivity than RT-PCR (Kishigami et al., 2014; **Figure 3D**).

**Table 3:** Detection of viruses from infected apple and pear shoots before and after incubation at high temperature (HT).

Experiment	Fruit tree	Virus	No. of plants detected ALSV by RT-PCR		
			
			Before HT^∗^	Just after HT	After growth at 25°C for 2 months
1	Apple^∗∗^	ALSV ACLSV	9/9 9/9	0/9 0/9	0/9 9/9
2	Apple	ALSV	7/7	0/7	0/7
3	Pear	ALSV	7/7	0/7	0/7
4	Pear	ALSV	8/8	0/8	0/8


*^∗^Apple seedlings were grown at 25°C and then at HT (37°C) for 4 weeks. After that, the seedlings were grown at 25°C for 2 months. ^∗∗^Apple plants infected with a mixture of ALSV and ACLSV.*

**FIGURE 4:**
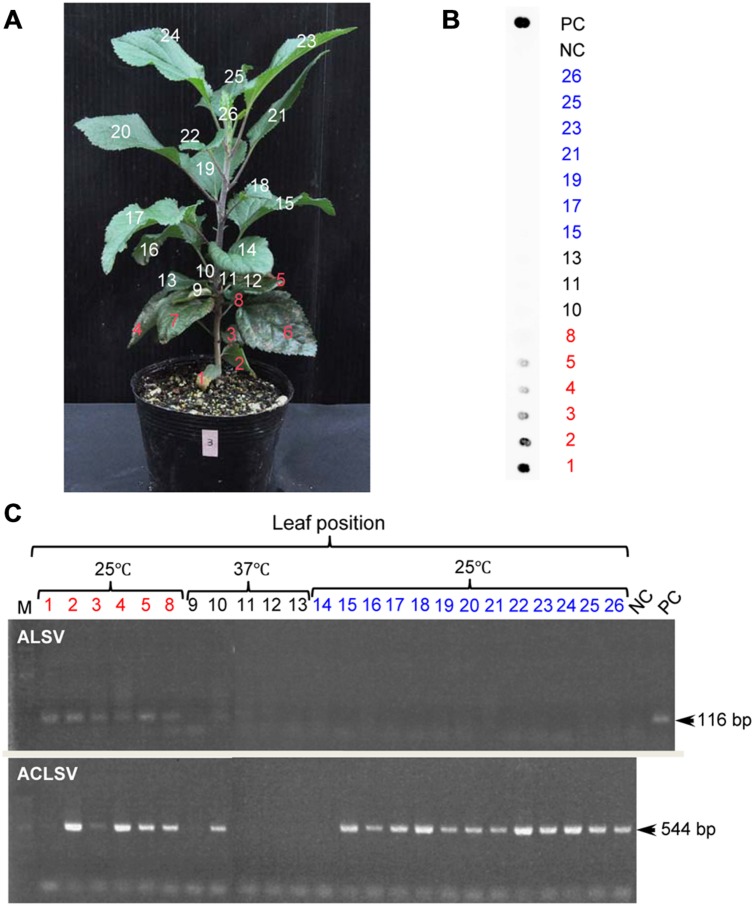
**RT-PCR detection of ALSV and ACLSV from an apple seedling after high-temperature treatment.** **(A)** Apple seedling grown at 37°C for 4 weeks followed by 25°C for 2 months. **(B)** Dot-blot hybridization of numbered leaves from the apple seedling in **(A)**. **(C)** RT-PCR detection of ALSV and ACLSV from numbered leaves from the apple seedling in **(A)**.

**FIGURE 5:**
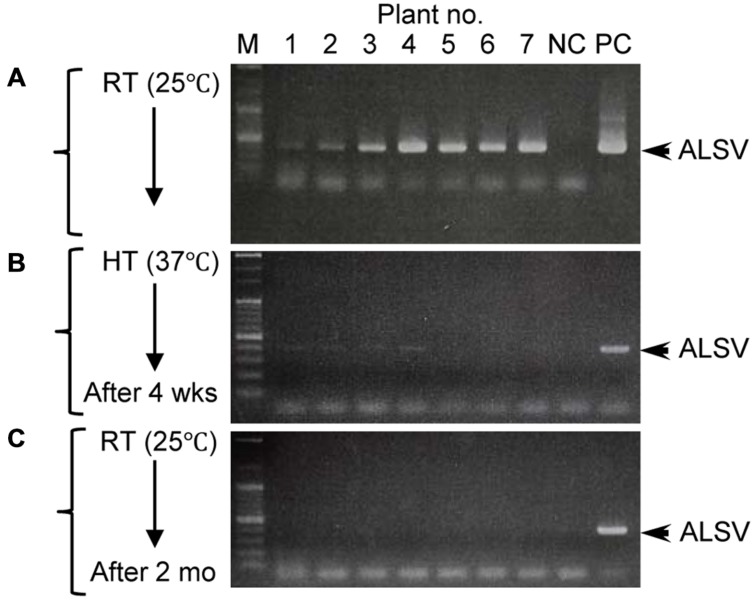
**RT-PCR detection of ALSV from pear seedlings before and after high-temperature treatment.** **(A–C)** Detection of ALSV from pear seedlings grown at 25°C **(A)**, then grown at 37°C for 4 weeks (wks; **B**), and transferred to 25°C and grown for 2 months (mo; **C**).

Although growth of apple plants was considerably reduced under high-temperature conditions (37°C), they grew rapidly once transferred back to 25°C (**Figure 4A**). We collected all leaves from apple seedlings grown for 2 months at 25°C after a 4-week incubation at 37°C, using dot-blot hybridization and RT-PCR to examine whether the virus had replicated (**Figures 4B,C**). ALSV was detected in leaves that developed prior to the high-temperature treatment (**Figures 4A–C**: 1–8). Conversely, neither dot-blot hybridization nor RT-PCR detected bands indicative of ALSV multiplication in the leaves that grew during the 37°C treatment (**Figures 4A–C**: 9–13) or in the leaves that developed thereafter at 25°C (**Figures 4A–C**: 14–26). In contrast, the control virus ACLSV exhibited different behavior, as it was detected in all leaves grown after being transferred to a 25°C environment (**Figure 4C**). In order to investigate the time required to inhibit the systemic movement of ALSV throughout the plant, the top leaves were assayed by RT-PCR 1, 2, 3, and 4 weeks after starting the 37°C incubation. The virus detection rate declined over time, leading to the conclusion that 4 weeks is the optimum duration for the elimination of ALSV (data not shown). The same high-temperature treatment of pear seedlings inoculated with ALSV followed by examination of viral movement to the whole plant body revealed inhibition of the movement of the virus throughout the plant, as in the case of apple seedlings (**Figure 5**, **Table 3**). Because apple and pear seedlings of different cultivars are usually propagated via grafting, the use of a shoot uninfected by ALSV vector should allow easy propagation of plants that are free of ALSV infection.

### *In Situ* Hybridization of Shoot Apical Meristem (SAM) of ALSV-Infected Apple and Pear Plants Before and After Incubation at High Temperature

**FIGURE 6:**
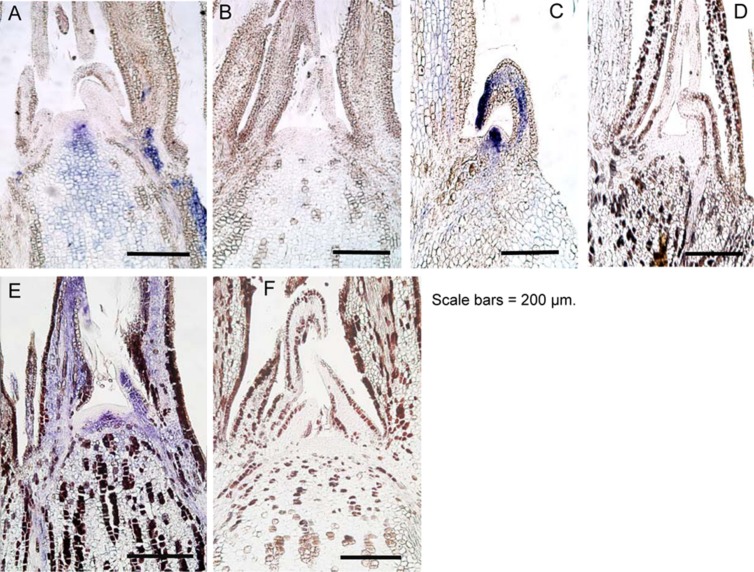
***In situ* hybridization of shoot apical meristem and lateral buds of apple and pear seedlings infected with ALSV.** **(A)** and **(C)**, apple seedlings grown at 25°C. **(B)** and **(D)**, apple seedlings grown at 37°C for 4 weeks followed by 25°C for 4 months. **(E)** Pear seedling grown at 25°C. **(F)** Pear seedling grown at 37°C for 4 weeks followed by 25°C for 4 months.

ALSV can invade the SAM of infected plants (Yamagishi and Yoshikawa, 2011; Yamagishi et al., 2011; Tamura et al., 2013). Characteristics of ALSV infection include a tendency for the virus to distribute itself homogenously throughout the plant. This is likely because ALSV moves from the SAM to the leaf primordium as soon as it replicates, in order to spread throughout leaf tissues (Yamagishi et al., 2011; Tamura et al., 2013). Thus, we investigated the distribution of ALSV in the SAM of apple and pear after high-temperature treatment using *in situ* hybridization. In contrast to the observation that signals indicating the presence of ALSV were strongly detected in the shoot apical tissue prior to high-temperature treatment (**Figures 6A,E**), no signals indicating ALSV multiplication and distribution were detected in the shoot apex, including the SAM of apple and pear grown at 25°C for 4 months after high-temperature treatment (**Figures 6B,F**). We also investigated ALSV distribution in apple lateral buds. While intense signals were detected in the lateral buds of apple plants not subjected to high-temperature treatment (**Figure 6C**), no ALSV signal was detected in the lateral buds of apple seedlings grown at 25°C following high-temperature treatment (**Figure 6D**). Thus, ALSV did not invade the SAM of the upper leaves of apple and pear plants grown at 25°C for 4 months following incubation at a higher temperature (37°C).

## Discussion

Since the start of agriculture, humans have advanced the selective breeding of crops toward a variety of goals, including the improvement of quality, increase in yield, and resistance to damage by diseases and insects. These types of breeding have primarily been performed using traditional cross-breeding (Hartung and Schiemann, 2014). Technologies directly transferring target genes into plants using recombinant DNA technologies came into use in the 1990s. However, the cultivation of genetically modified plants produced using recombinant DNA technology is restricted by laws regulating the use of genetically modified organisms (such as the Cartagena Protocol in Japan; Hartung and Schiemann, 2014). In addition, new plant breeding methods are under development that use recombinant DNA technology, but makes it possible to leave no trace of introduced DNA in the genome of the final product (a new variety, for instance; Hartung and Schiemann, 2014).

ALSV vectors are useful viral vectors that can be applied to various plant species, including *Rosaceae* fruit trees (Li et al., 2004; Igarashi et al., 2009; Yamagishi and Yoshikawa, 2009, 2011; Sasaki et al., 2011; Yamagishi et al., 2011, 2014; Takahashi et al., 2013; Taki et al., 2013; Tamura et al., 2013; Kon and Yoshikawa, 2014; Satoh et al., 2014; Nakatsuka et al., 2015). As mentioned, the improved ALSV vector includes three additional multi-restriction enzyme sites (**Figure 1A**), and allows up- and down-regulation of expression of multiple genes concurrently. Yamagishi et al. (2014) developed a next-generation technology that can trigger the flowering of apple seedlings about 2 months after germination, which allows for the production of next-generation seeds within a year. This is accomplished by infecting apple seedlings with ALSV-AtFT/MdTFL1 concurrently expressing the *A. thaliana* florigen gene (*AtFT*) and suppressing the expression of the apple anti-florigen gene (*MdTFL1-1*; Yamagishi et al., 2014). If this technology becomes available for other fruit trees with a long juvenile period, it would be very useful for the breeding of fruit trees.

In this study, we described how ALSV-AtFT/MdTFL1, which is effective at promoting flowering and shortening the generation time of apple, also promotes flowering in pear. Moreover, we confirmed that ALSV-AtFT/PcTFL1, into which part of the pear *PcTFL1-1* gene has been introduced, induces early flowering at a high rate, after which seedlings undergo continuous flowering for at least 2 months. The subsequent formation of fruits was also confirmed (**Figure 2**). In other words, ALSV vector technology is applicable to fruit trees besides apple. However, the ALSV systemic infection rate was very low in Japanese pear compared to pear and apple (**Table 1**). Because ALSV could replicate in inoculated leaves of Japanese pear, the low systemic infection rate was likely due to inhibition of viral movement and/or inactivation of the virus.

Regarding early flowering in pear, it has been reported that genetically modified plants expressing the *FT* gene and genetically modified plants suppressing the *TFL* gene both exhibit early flowering (Matsuda et al., 2009; Freiman et al., 2012). However, these genetically modified plants cannot be used for breeding according to the Cartagena Protocol mentioned above. Because the breeding of fruit trees such as apple and pear uses vegetative propagation in most cases, successful and easy elimination of the virus from a plant previously infected with an ALSV vector allows the plant itself to be used as breeding stock.

Currently available techniques for the elimination of viruses from infected plants include thermotherapy, chemotherapy, meristem culture, and techniques combining these technologies (Panattoni et al., 2013). Thermotherapy is the most frequently used method for obtaining virus-free tissue, whereas in fruit trees such as apple, it is most common to culture the shoot apex tissues, where no virus is distributed, after heat treatment (Knapp et al., 1995; Bhardway et al., 1998; Valero et al., 2003; Paprstein et al., 2008; Wang et al., 2008; Tan et al., 2010; Hu et al., 2014). In this study, we demonstrated that high-temperature treatment of ALSV-infected plants inhibits the systemic movement of ALSV, enabling virus-free shoots to be obtained (**Table 3**, **Figures 3–6**). For example, when apple infected with both ALSV and ACLSV was incubated for 4 weeks at 37°C and then grown at 25°C, ACLSV began replication in the new leaves although virus could not be detected just after high temperature treatment, but ALSV did not invade the new tissues (**Figures 3–6**). It is likely that incubation at high temperature inhibits systemic movement of ALSV because it becomes difficult to invade tissues derived from the SAM once excluded from the SAM.

**FIGURE 7:**
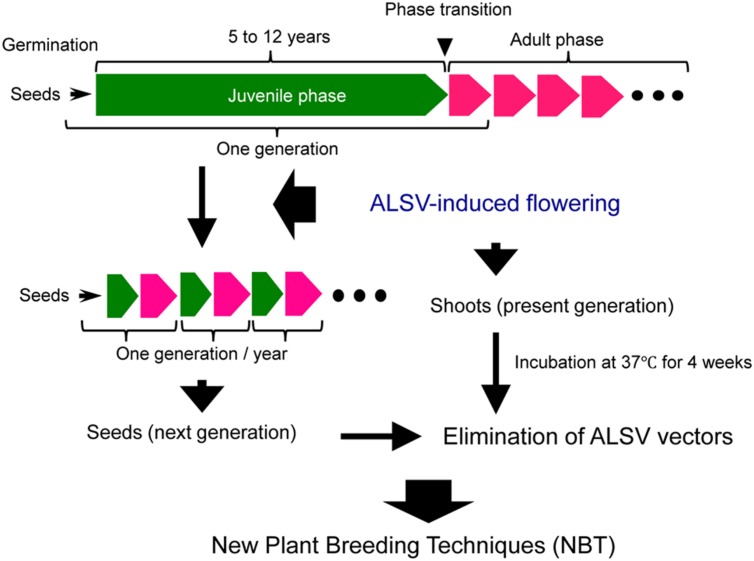
**Application of ALSV vector technology, a new plant breeding technique for the accelerated breeding of fruit trees**.

As mentioned above, the use of ALSV vector technology allows advancing the timing of flowering in apple and pear to several months after germination, thus shortening the generation time to 1 year or less (**Figure 7**). A great convenience is that most of the next-generation seedlings obtained from the apple plants exhibiting early flowering were ALSV-free. In addition, ALSV could easily be eliminated from infected apple and pear individuals using a high-temperature treatment of 4 weeks at 37°C (**Figure 7**). Because the ALSV vectors are not transformed into the genome of plant, the plants after the high temperature treatment would be considered non-transgenic and would not fall under the biotechnology regulations. In other words, we consider ALSV vector technology to be very effective as a new plant breeding technique for fruit trees, because the final products (next-generation seedlings or high-temperature-treated plants) do not fall into the category of genetically modified plants, even though the technology uses a recombinant virus to trigger early flowering in apple and pear seedlings.

## Author Contributions

CL constructed the ALSV vectors used in this study. NYa carried out the inoculation of the ALSV vectors to apple, pear, and Japanese pear and RNA analysis. NYo is the principal investigator, supervised the experiments and the writing of the manuscript.

## Conflict of Interest Statement

The authors declare that the research was conducted in the absence of any commercial or financial relationships that could be construed as a potential conflict of interest.

The reviewer HF and handling Editor declared their shared affiliation, and the handling Editor states that the process nevertheless met the standards of a fair and objective review.
